# Because its power remains naturalized: introducing the settler colonial determinants of health

**DOI:** 10.3389/fpubh.2023.1137428

**Published:** 2023-07-11

**Authors:** Bram Wispelwey, Osama Tanous, Yara Asi, Weeam Hammoudeh, David Mills

**Affiliations:** ^1^Department of Global Health and Population, School of Public Health, Harvard University, Boston, MA, United States; ^2^Division of Global Health Equity, Department of Medicine, Brigham and Women's Hospital, Boston, MA, United States; ^3^François-Xavier Bagnoud Center for Health and Human Rights, School of Public Health, Harvard University, Boston, MA, United States; ^4^School of Global Health Management and Informatics, College of Community Innovation and Education, University of Central Florida, Orlando, FL, United States; ^5^Institute of Community and Public Health, Birzeit University, Birzeit, Palestine; ^6^Division of Emergency Medicine, Boston Children's Hospital, Harvard Medical School, Boston, MA, United States

**Keywords:** settler colonialism and native dispossession, indigenous health, health equity, structural racism, logic of elimination, social determinants

## Abstract

Indigenous people suffer earlier death and more frequent and severe disease than their settler counterparts, a remarkably persistent reality over time, across settler colonized geographies, and despite their ongoing resistance to elimination. Although these health inequities are well-known, they have been impervious to comprehensive and convincing explication, let alone remediation. Settler colonial studies, a fast-growing multidisciplinary and interdisciplinary field, is a promising candidate to rectify this impasse. Settler colonialism’s relationship to health inequity is at once obvious and incompletely described, a paradox arising from epistemic coloniality and perceived analytic challenges that we address here in three parts. First, in considering settler colonialism an enduring structure rather than a past event, and by wedding this fundamental insight to the ascendant structural paradigm for understanding health inequities, a picture emerges in which this system of power serves as a foundational and ongoing configuration determining social and political mechanisms that impose on human health. Second, because modern racialization has served to solidify and maintain the hierarchies of colonial relations, settler colonialism adds explanatory power to racism’s health impacts and potential amelioration by historicizing this process for differentially racialized groups. Finally, advances in structural racism methodologies and the work of a few visionary scholars have already begun to elucidate the possibilities for a body of literature linking settler colonialism and health, illuminating future research opportunities and pathways toward the decolonization required for health equity.

## Introduction

“Wholistic approaches to Indigenous health that acknowledge political, social, cultural, spiritual and economic contexts remain at the margins of current health-care practices and frameworks. Although land is broadly considered to be a determinant of health, it has rarely been incorporated into health policy and practice.”

-Lana Ray et al. (2019) (1).

The quest to elucidate the “causes of the causes” of health outcomes has recently returned in force to health sciences discourse in the Global North (2). While often still enduring the misguided or overblown explanatory frameworks of racial essentialism (3), eugenics (4), biomedical fixation (5), genomics (6), individualism (7), cultural and behavioral primacy (8, 9), and neoliberal victim-blaming (10), academic health sciences are taking greater notice of social structures and their determinant health ramifications (11), thanks in part to mass social movements (12). As one healthcare journal recently put it with the launch of its social determinants of health newsletter, these determinants are “having a moment” (13). Marking a resurgence of Virchow’s 19th century social medicine theory (14), this development represents an epistemic vindication of much North American and Australasian Indigenous (15), Black (16), Palestinian (17), Latin American (18), and other scholarship from marginalized communities and voices, many of whom have been scholars and activists residing in the academy’s—or broader society’s—undercommons (19–21).

Structural competency is now widely recognized as an essential component of good clinical practice, effective pedagogy, and reliable research (22). Structural determinants are upstream and explanatory of the even more widely grasped social determinants of health (23, 24), which include conditions like poverty, education, homelessness, and the degree of access to healthcare. Without a structural framing, however, the etiologies of social determinants are widely interpretable and thus deployable for a range of political and policy ends, both racist and antiracist. Recognizing the importance of such a framing, the World Health Organization’s Committee on the Social Determinants of Health (CSDH) framework released a report stating that the most upstream structural factors are the socioeconomic and political contexts, which include governance, macroeconomic policies, social policies (e.g., labor market, housing, land), public policies (e.g., education, health, social protection), and culture and societal values (25). Following these are the social determinants (e.g., social class, gender, race) and what CSDH terms intermediary determinants, which include a grab-bag of components such as material living circumstances and psychosocial, behavioral, and biological factors. Regardless of the specific framework referenced, and any quibbles one may have with the details, structural approaches have, despite increasingly incisive calls (26–29), left out something crucial for elucidating health inequities: settler colonialism.

The persistence and degree of Indigenous health inequities, and the lack of conceptual clarity around what is driving them, suggest this has been a consequential exclusion. In this article, we argue that the adoption of a settler colonial analytic in Indigenous public health research and interventions provides a means to rectify these deficits in the understanding of health inequities, as well as the possibility for more productive approaches to their amelioration. As with structural racism, a focus on settler colonialism “offers a concrete, feasible, and promising approach toward advancing health equity and improving population health” (11). The goal of the article is therefore twofold: to provide a conceptual argument that settler colonialism shapes health in ways that are both fundamental and distinct from other determinants, and to demonstrate that this relationship is observable and testable with commonly accepted methodologies.

While the article’s focus is the health impacts of settler formations and strategies, the scholarship and insights informing it reflect predominantly Indigenous perspectives on their historical and ongoing relationships to both settler colonialism and Indigeneity.^1^ Kauanui reminds us that settler colonial studies “does not, should not, and cannot replace Indigenous studies” (30), and just as the most insightful work on anti-Black structural racism and health includes engagement with critical race theory and Black studies in addition to society’s manifest racism, a settler colonial health analytic must engage both of the interrelated but distinct disciplines focusing on settler colonialism and Indigeneity. Well-executed research and interventions to address the settler colonial determinants of health will by necessity be grounded in Indigenous studies, experiences, perspectives, and resistance, particularly as Indigenous health priorities and conceptualizations may often differ from those of settler society and its health representatives (31). In this sense, the settler colonial determinants of health, which can also provide insights into the health of non-Indigenous populations, might best be categorized within the larger umbrella of Indigenous health determinants.

### Settler colonialism: a brief introduction

A social formation structuring nation-states like the United States (US), Canada, Australia, New Zealand, and Palestine/Israel,^2^ settler colonialism exists as the “inherited background field” (32) within which other “interlocking systems of oppression” (33) – racism, patriarchy, economic extraction, etc. – converge to shape structural health determinants in these societies. Settler colonialism is a form of exogenous domination in which the primary goal of the colonial movement is to obtain and stay on the land, seeking the elimination of Indigenous^3^ communities—as individuals but especially as peoples with sovereign status and claims—rather than their exploitation for labor and trade as in franchise colonialism^4^ (e.g., the Dutch East Indies or British India) (35). This Native displacement is in the service of a settler replacement that generally mirrors the metropolitan homeland(s), and often includes genocidal settler violence of a sort that is rarely seen in franchise colonial settings where it would compromise resource extraction (36, 37). Settler society then structures itself into “a relatively secure or sedimented set of hierarchies that continue to facilitate the *dispossession* of Indigenous peoples of their lands and self-determining authority” (32). These hierarchies are produced and maintained through a variety of gendered (38), racialized, and economic means, and settler societies often devise more participatory and democratic (if still Native-exclusionary) societies than existed in their origin countries in order to maximize cross-class settler cohesion in the face of inevitable Indigenous resistance.

Unlike immigrants, who must abide by the customs and laws of existing communities, settlers^5^ “carry their sovereignty with them” (39), creating separate polities and justifying the expropriation of Native land through the racialized narratives of superiority that are a hallmark of Western modernity and racial capitalism (40–42). In Mamdani’s words, “[s]ettlers are made by conquest, not just by immigration” (43), or as he later phrased it, “[t]he history of creating new polities atop preexisting societies is…what distinguishes settlers from immigrants” (44). It is worth noting that while this distinction may matter very much to those arriving in a new location, it may be less meaningful to the Native inhabitants experiencing ongoing dispossession and displacement (45, 46). For nation-states like the US or Australia, it is impossible to imagine their basic demographic make-up and political structures without the settler colonial social formations that precede and continue to form them. Settler colonialism is not a remote and potent event, a Big Bang of settler states’ history, but is instead an enduring structure actively shaping the present (47). This continued settler colonial presence has exploded beyond settler borders to have a profound influence on social, economic, and political structures across the globe today, in part through the construction of modern democratic thought (48–50). This is in no small part because the world’s first settler colonial state, the US (51), is its most powerful, wealthy, and socio-culturally dominant, continuing to enact “reiterations of [its] pioneer logics” in its interventions and influence around the world (52).

Settler colonialism is not only distinct from, if often intertwined with, franchise colonialism’s status as an “external, extractive relationship between a colonizing metropole and a colonized periphery” (53, 54). In settler colonialism’s most successful manifestations, they are closer to opposites: settlers seek to replace and ultimately *become* “indigenous,” collapsing the colony and metropole spatially and conceptually. For this reason, in places like the US or Australia where Indigenous people are often explicitly written out of history and modernity even while vibrantly present (34), settler colonialism is indiscernible to settler society and replaced by the purported postcolonial existence of a newly imagined nation-building project (48, 51, 55). Settler colonialism “covers its tracks” by orchestrating its own demise, removing and otherwise making invisible the remaining colonized people such that it can disavow any colonial existence and peddle narratives of an immigrant and multicultural society in which no groups have distinct sovereignty claims (44, 53). This contrasts with franchise colonialism’s need to reproduce itself, maintaining a clear distinction between the colonizer and the colonized, whose valuable labor ensures they remain subordinately present and visible. Stressing settler colonialism’s distinction from franchise colonialism is not simply an academic exercise; it has crucial implications for decolonization. While it is known how formal decolonization of franchise colonial contexts proceeds conceptually and empirically (36), at least into a neocolonial phase (53), decolonizing settler societies requires a radically different and as yet unborn process (44, 56, 57). A correct diagnosis is essential if there is to be hope for a cure.

It may be helpful to think of current settler nation-states dialectically, as exhibiting variable degrees of perpetration of the settler colonial project as well as a range of historical and ongoing forms of Indigenous resistance in opposition. The reasons for different degrees of settler invasion are myriad and deeply influenced by “preaccumulation,” or the historical endowment of resources drawn from the metropolitan population that have been available to settlers in overcoming Native resistance (58, 59). Furthermore, the settler colonies most dependent on Indigenous labor–i.e. representing a mix of franchise and settler colonialisms – are those that have been most successfully challenged by mobilized Indigenous movements, as in Algeria, South Africa, Kenya, and Zimbabwe (Rhodesia), among others (60). If the US and Australia inhabit one pole of demographic and territorial attainment with frontier closure around clearly demarcated reservations and homelands, at the other are the Native movements in Algeria, Kenya, and Zimbabwe that thwarted settler colonial invasions and associated attempts at achieving a discrete and hegemonic settler polity. A spectrum lies between these two poles, including contexts like Western Sahara, Northern Ireland, and Palestine/Israel. While settler colonialism achieves something close to invisibility or deniability at the first pole, it becomes more discernible where it is most imperfect and unsuccessful toward the second (53). While “organized violence” is still active at the first pole through policing and incarceration (61), settler colonial dispossession is no longer driven primarily by expansionary state violence but rather by a governmentality that dictates Indigenous recognition and accommodation (32). It is worth noting that the closing of the frontier, as has occurred in the US, is not synonymous with completion of the settler colonial project: creative and indomitable Native resistance and resurgence continue in these geographies today (62–66) despite legal setbacks led by state legislatures, treaty-violating executive actions, liberal processes of Indigenous recognition (32), and sovereignty-denying, trans-partisan Supreme Court decisions (67, 68).

### The public health implications of a settler colonial analytic

Despite the rich and rapidly expanding social science literature conceptualizing settler colonialism, “this research has not been adequately integrated into medical and scientific literature geared toward clinicians and other health professionals,” as Bailey and colleagues similarly note in regard to structural racism (11). Settler colonialism is to the structural determinants of health as the structural are to the social, fundamentally reshaping the socioeconomic, political, and land-based environments through Indigenous erasure and settler hegemony. As the mode of domination that innervates the structures that overdetermine health (24), settler colonialism also shapes the health systems that are imposed separately on Indigenous peoples in forms that are limited and understaffed such that (69), in their failures, they serve to justify further colonization and sedimentation of hierarchies (70). The degree and specific manifestations of settler colonial encroachment and expropriation relate directly to the Indigenous health impacts. While broadly analogous across contexts, health outcomes inevitably express a specificity that reflects colonial chronicity and the local dialectics of settler invasion and Indigenous resistance. Where expansionary attempts are ongoing, as in the case of Palestine/Israel, the outcome is active settler frontier violence, forced displacement, and the inevitable health consequences that follow (71). In a world where humanitarian values and human rights are lauded and overt Native elimination is illegitimate, frontier violence has in many cases transformed from its original North American and Australasian ferocity to population fragmentation (72), village and property destruction (73), maiming (74), unchilding (75), spatial sequestration (35), and more concealed forms like gentrification (76), assimilation (35) and liberal modes of recognition (32). As we discuss later, the acute and chronic health impacts of such settler colonial technologies and strategies can be observed and analyzed with primary research, and additional modes of elimination can be theorized from Indigenous and settler colonial studies and likewise evaluated.

While a strong grasp of history is no doubt essential for elucidating the “causes of the causes” of health inequities, it is the above-mentioned contention – settler colonial invasion is an enduring “structure not an event,” in Wolfe’s famous phrasing (47) – that generates the possibility of theorizing the settler colonial determinants of health. Channeling North American Indigenous and Palestinian thought and scholarship stretching back decades (30, 77), Wolfe’s unveiling of the settler colonial present, along with the contention that settler colonialism follows a logic of Native elimination^6^ (35), stimulated settler colonial theory’s remarkable ascendance over the last two decades. Veracini describes Wolfe’s axiom as having “kick-started what with hindsight could be called the ‘settler colonial turn,’ the phrase that, by a very conservative estimate, launched a thousand papers” (48). This growing wave of interdisciplinary scholarship is now reaching the shores of public health (28, 71, 72, 79–85).

### Understanding indigenous health inequities: the opportunity and the challenge

What might the convergence of the fields of settler colonial studies and public health help us accomplish? Indigenous people suffer earlier death and more frequent and severe disease than their settler counterparts, a remarkably persistent observation over time, across settler colonized geographies (86–88), and despite ongoing efforts to resist their elimination. While these health inequities are well-known, they have been relatively impervious to comprehensive and convincing explication in the public health and medical literatures (89), let alone remediation. Instead, comparative Indigenous health data have more commonly been used to justify and rationalize past and present colonial modes of domination (81, 90). Epidemics were exploited by North American settlers, often with the collusion of health professionals, to obtain land and to “civilize” and assimilate Native peoples (91). Permeated by the logics of Western modernity and colonialism, scientists and physicians continue to utilize health inequities to concoct and reinforce a transcontinental myth of Native biological inferiority and fragility (53, 92), what Farmer would call an “immodest claim of causality” (93).

The consistency of health inequities among geographically and genetically divergent Indigenous peoples across settler colonial contexts should lead us instead to a radically different explanatory model, shifting the focus – and the culpability – from Native biology and culture to settler sociopolitical formations and their attendant violence. While this shift risks reinscribing settler centrality and importance, it is a necessary but insufficient corrective to an otherwise essentialized or pathologized Indigeneity. A further reckoning with Indigenous studies and Kauanui’s conception of “enduring Indigeneity” remains imperative for any investigation into the health ramifications of settler colonialism (30). Successfully making the transition to an Indigenous guided appraisal of settler responsibility requires a testable framework dealing in historical and social structures rather than racist and pseudoscientific tropes. Theories and frameworks like ecosocial theory (94), structural racism (11), and Public Health Critical Race praxis (95) are exceptionally comprehensive, but settler colonial theory provides something additive: the overarching eliminatory logic that explains *why*—and might yet help us better understand *how*—White supremacy and structural racism are embedded within settler policies, institutions, laws, academia, and societal practices, from there becoming embodied in Indigenous and other racialized non-settler peoples (e.g., “arrivants” (52, 96)). Settler colonial theory also incorporates issues of sovereignty and land, clarifying health-impacting mechanisms that are specific to Native inhabitants. If we grasp settler strategies and the structurally-desired endgame—the elimination of Indigenous societies as sovereign and Native communities and individuals as capable of resisting and thriving—a more constructive approach can be devised in the name of health and human flourishing.

Claiming settler colonialism as the principal upstream explanator of settler-Indigenous health inequities still begs a closer inspection of precisely how this societal formation maintains such predictably toxic effects on Native peoples, particularly if this framework is to become widely accepted within public health and epidemiology. Given ideological barriers and widespread education deficits in Indigenous history, some have suggested that incorporating settler colonialism into health models may be better addressed by a major overhaul of these disciplines. Qato notes that, rather than an unthinking reliance on “ever-more sophisticated critical methodologies, theories, or tool kits,” achieving the transformative potential of public health requires

challenging the logics of research itself, the epidemiological models upon which this research is built, and the data upon which policies are enacted and imagined. We must push back against the stubbornly dominant frames of individuated biomedical interventions that attempt to neutralize the political praxis underlying any robust and effective public health response. In contrast to the abundant research mapping prevalence of health outcomes and deploying ostensibly definable and quantifiable explanatory variables, there has been little substantive exploration, if any, of what it would mean to incorporate settler colonialism into our models of health (79).

The existence of a causal connection between settler colonialism and health may seem clear enough to those accustomed to structural analytic framings, if still diffuse and vague (83). Venturing such a discursive claim of certainty while taking aim at the scientific peddlers of racism and biological determinism, Wolfe launched the settler colonial line of health inquiry with a rhetorical question back in 2006: “Even in contemporary, post-Native Title Australia, Aboriginal life expectancy clings to a level some 25% below that enjoyed by mainstream society, with infant mortality rates that are even worse. What species of sophistry does it take to separate a quarter “part” of the life of a group from the history of their elimination?” (35). Execrable sophistries aside, the rigorous evidence needed to connect that missing quarter life portion to the past and present ravages of settler colonialism is still largely incomplete. As Krieger reminds us, “…it is one thing to observe an association. It is another to explain it. This is why theory, causal assumptions, and frameworks are key, not just the observable ‘facts’” (97). Qato echoes Wolfe’s certainty regarding causal assumptions and reinforces Krieger’s bracing task ahead when she claims that “settler colonialism precedes and is fundamental to all other determinants of health—be they clinical, economic, social, or political” (79).

While some social scientists might already concur with the contention that settler colonialism and health are both obviously and powerfully linked, it is epidemiologists and public health specialists who often decide what counts as causative health “evidence” and can thus become eligible for population-level knowledge consumption. Such a gatekeeping role, infused with and often blind to coloniality (98), has historically positioned these disciplines as complicit in perpetrating epistemic violence and preservation of the status quo (99–101). Rewiring our health models and research questions from a starting point of settler colonialism is an opportunity to challenge the epistemicide of Native and other colonially marginalized voices that exists as the gaping wound at the heart of these disciplines’ underlying assumptions (102).

Given this undergirding coloniality, it is tempting to suggest that compelling, data-driven explanations linking settler colonialism to health are, with currently available research tools and historical access, undiscoverable or perhaps even unhelpful. While standing behind data’s transformative potential, Krieger warns of the dangers of problematic use of data on racialized groups, for example, recognizing that data has been readily used as a tool of oppression and does not inherently move us closer to clarifying or addressing injustice (103). TallBear notes how the interest in Indigenous heredity exhibited by the field of genetic science represents the latest attempt to determine and control Native identity for settler advantage (104). Regarding theoretical challenges with upstream explanators, De Maio and Ansell describe the limitation of utilizing Galtung’s concept of structural violence (105), which has been invoked as an “overarching explanatory framework” for health inequity but typically in terms that are broad, vague, and unsuitable for empirical testing (106). Beyond the challenges of testability, the primary problem with structural violence, they note, is a lack of specificity regarding origins and perpetrators, something fixed by merging the concept with a larger theoretical framework like “critical race theory, feminism, Marxism, or *other approaches*” [emphasis ours] (106). Its role in incisively excavating the origins and perpetrators of violence is precisely where settler colonial theory is most valuable, expressing a conceptual precision as one of these “larger theoretical frameworks.” Yet, in the most ambitious attempt to explicate settler colonialism’s health impacts, Paradies braces us for the gloomy possibility of data’s elusiveness in suggesting that “[a]lthough colonization may be the “root cause” of indigenous ill health, its aftermath may not be directly measurable in an epidemiological sense” due to “deep epistemological challenges in tracing the impact of colonization over centuries” (83). He also wonders “[w]hat analytical purchase does [colonialism] add, if any, to the existing body of research on racism and indigenous health?” (83).

In making the case for a settler colonial analytic in health research and interventions, this paper seeks to directly address these questions and concerns. Despite the relative paucity of research to date, we offer three arguments that together constitute an initial theorizing of the settler colonial determinants of health. While still premature, such an undertaking is intended to be propaedeutic and may prove useful for those hoping to investigate the mechanisms connecting settler technologies and techniques to health outcomes. A first step is to reject the idea that we are only or even primarily dealing with an “aftermath” (83), which indicates a conflation of the two primary types of colonialism in implying a postcolonial existence that has not yet come for Indigenous people still resisting settler colonialism’s enduring reality (47, 63). This settler colonial present is the topic of Argument One, in which we explore some of the manifestations, techniques, and technologies of elimination that are well-suited for testable study on health outcomes. While we rely primarily on examples from the places where we live and work—Palestine/Israel and the US—there are several other settler colonial contexts, which, despite regularly borrowing technologies and techniques from each other, will have their own specific elements. Whether racism, is sufficient for understanding and analyzing settler colonialism’s ongoing effects, is the subject of Argument Two. Argument Three focuses on emerging scholarship that is characterizing the health impacts of settler colonialism as well as potential methodologic paths forward to concretize this linkage (See Box 1 for argument summaries). The concluding Discussion returns us to the purpose of theorizing the settler colonial determinants of health and interrogates what might be required to mitigate or abolish them.

## Argument

### Argument i. Elimination and health in the settler colonial present

“Settler colonialism is woven, in ways both known and unknown, into these [clinical, economic, social, and political health] determinants. In its direct attacks on us and on the environments in which we live and seek care, settler colonialism distorts our relationships with our bodies.”

-Danya Qato (2020) (79).

“Settler colonialism is territorially acquisitive in perpetuity.”

-Glen Coulthard (2014) (32).

In “Colonization, Racism, and Indigenous Health,” Paradies interrogates historical trauma as the primary existing model developed to understand the present-day effects of colonization on health (83), noting a lack of coherent evidence to date (107) and that much existing theory on historical trauma depends on a clear demarcation between past and present. Following Indigenous scholars like Gone in characterizing this distinction as potentially distorting and essentializing (108), Kirmayer and colleagues offer a framework to understand historical trauma as ongoing structural violence impacting mental health while acknowledging that “[e]stablishing causal linkages across generations in the case of historical trauma \is exceedingly difficult, perhaps even impossible” (109). While this challenge stems from the fact that “mental health problems are common, multiply-caused, and nonspecific,” the authors note that transgenerational impacts of the Holocaust have been linked in studies to depression, anxiety, and post-traumatic stress disorder (109). But these impacts attenuate or disappear over two or three generations, while this is often not the case with North American and other Indigenous descendants, suggesting “more proximate causal factors must predominate to account for this increased incidence of suffering within contemporary populations” (109). Drawing on the work of psychiatrist Frantz Fanon, Coulthard locates these proximate factors within settler colonial society’s *ongoing* interpellation of Indigenous people, generating internalized racism and associated symptoms of low self-esteem, depression, and maladaptive coping mechanisms (32).

In order to theorize settler colonial health determinants that can be observed, studied, and intervened upon, it is necessary to make the argument, stressed in the introduction, that settler colonialism is alive and well. Much of this theoretical and descriptive work has been accomplished (30, 32, 47, 48), and the editorial statement from the journal *Settler Colonial Studies* remarks that “[t]here is no such thing as neo-settler colonialism or post-settler colonialism because settler colonialism is a resilient formation that rarely ends” (110). In other words, settler colonialism has active contemporary, and thus prospectively researchable, manifestations.^7^ Even if we accept that the epidemiological challenges of linking present Indigenous illness to historical events may be insurmountable, the settler colonial present provides a clear path forward: the “proximate causal factors” can still be explicated and framed within their current settler colonial contexts (109). At a conceptual level, then, the difficult task of attempting to connect remote events to present health conditions is not the impenetrable barrier to analyzing settler colonial determinants of health that Paradies’ questioning might suggest. In addition to resisting futility when it comes to better describing the past’s impact on the present, we can simultaneously ask questions about settler colonialism’s contemporary manifestations in ways that are testable with widely accepted methodologies. The first step is to describe these technologies, techniques, and manifestations, recently designated in the Palestinian context as “strategies of elimination” (111), and then draw up appropriate questions to examine their health impacts as one would with any other research question.

While Paradies does briefly distinguish settler colonialism from other colonial forms, parts of his analysis suggest some degree of conflation or ambivalence about settler colonialism’s current role. This equivocation may be due, at least in part, to his lack of textual attention to a settler colonial frontier, that of Palestine/Israel (35, 44, 45, 57, 112–115), representing a more “unconcealed structure of domination” (32) than those currently present in Canada, the US, Australia, and New Zealand. Unlike these longer term cases, the Zionist settler movement dates only to the 19^th^ century, albeit still “in the context of an imperial common sense in which Europeans *could and should* settle everywhere” (116), and the mass expulsion of Palestinians occurred just as the global shift toward decolonization began. Today, one need only consider the commonness of terms like “Israeli settler” or “West Bank settlements” and to see their ongoing state-sponsored encroachments to grasp what is meant by a settler colonial present. This hyper-visible Palestine case thus provides a unique temporal lens for understanding settler colonial health determinants more broadly, including those in the above-mentioned Anglo-settler contexts. As with Palestinian experiences of ongoing land theft, dispossession, and the forced displacement of Native people, the most readily describable manifestations of settler colonialism are those driven by the logic of elimination. Elimination, which “should be seen as an organizing principle of settler colonial society rather than a one-off (and superseded) occurrence,” can take on myriad forms (45). Large-scale killing, once a hallmark of settler colonial conquest and frontier violence, is now only deemed acceptable under extreme conditions even in the prolonged states of exception generated by regimes wielding power over life and death (74, 117–119).

Eliminatory forms have therefore diversified since the first half of the 20th century with the advent of human rights discourse and the formal franchise decolonization of much the globe. These can include forced displacement, dispossession, spatial sequestration, movement restrictions, permit regimes, gentrification, settler self-indigenization, various forms of biocultural assimilation, maiming, and other excessive uses of force (76, 111, 120–122). Eliminatory techniques are mutually reinforcing and co-constitutive, although not all of them will be present in each settler context and time period. Rather than be prescriptive, our goal is to provide tools and examples, mostly from our contexts in Palestine/Israel and the US, with the understanding that local Indigenous knowledge and priorities should determine targets for investigation and intervention. Alone or together, each of these eliminatory techniques can be studied for their health effects, often through well-described social determinant intermediaries. Examples might include the impoverishment resulting from home demolitions and ongoing settler invasion (123), environmental exposures and lack of sufficient clean water related to displacement, limited healthcare access resulting from movement restrictions or sequestration (123, 124), and forced dietary changes through settler appropriation of agricultural land and initiation of diabetogenic rations and/or economic capture (125). Some eliminatory manifestations can also be analyzed for direct health impacts, including Israel’s refusal to administer COVID-19 vaccines to occupied Palestinians (126), limb loss from intentional maiming (127), psychosocial trauma resulting from settler violence, and stress-related inflammatory and hormonal dysregulation from institutional and interpersonal anti-Indigenous racism. Allostatic load, encompassing the cumulative burden of persistent inflammatory marker release and hormonal dysregulation in response to chronic stress, is one well-described pathway that can be readily investigated within a racialized settler colonial context (128). Many of settler colonialism’s manifestations, including physical displacement, cultural destruction, or anti-Indigenous discrimination, inevitably over-activate the physiologic stress pathways that are increasingly linked to chronic disease development in other racialized populations (11).

The American innovation of spatial sequestration, which impacts health through mechanisms like impoverishment, lack of employment, overcrowding, and limited healthcare access, among others, continues to have lasting transnational settler colonial influence. In the mid-19^th^ century, as the frontier east of the Mississippi dwindled, the US under presidents Lincoln and Grant implemented the reservation as a form of Native removal and enclosure, providing the model for South Africa’s Bantustans, Germany’s Namibian and European concentration camps, and the besieged Gaza Strip today (36, 51). The infamous calorie restriction planned by Israel for the Strip’s two million Palestinians, part of a strategy to keep the territory “on the brink of collapse,” is just one eliminatory tactic that sequestration facilitates (129, 130).

Spatial sequestration inevitably begins with forced displacement, a “disruption of Indigenous relationships to land [that] represents a profound epistemic, ontological, cosmological violence,” and with enormous ramifications for health and thriving as a result (131). This practice has impacted the majority of Palestinians worldwide, millions of whom live within a few dozen overcrowded refugee camps in Palestine/Israel and neighboring countries. The Gaza Strip itself exists as an artificially bounded territory populated predominantly by displaced Palestinians and their descendants. Forced displacement occurs today through Israeli land policies, including Supreme Court-sanctioned plans to ethnically cleanse West Bank villages in order to corral Palestinians into disconnected urban zones (132). In the Naqab, the state of Israel uses home and village demolitions in addition to the promise of health services to entice Palestinians from their lands into overcrowded cities and towns, suggesting that healthcare itself can become a tool of eliminatory sequestration (133). Enclosure is further enabled through the implementation of apartheid (“apartness” in Afrikaans) policies that intentionally maintain the domination of one racial group over another, as seen in Apartheid South Africa and Palestine/Israel today (42, 134, 135). Decades ago, such policies were recognized by the World Health Organization as incompatible with the right to health and its actual attainment (136).

Other forms of elimination became prominent in the 19th and 20th centuries, including the invention of American and Canadian boarding schools that sought to remove children from their parents in order to “kill the Indian…and save the man” (35, 137). While these schools attempted to erase entire cultures under the façade of humanitarianism and the “civilizing” mission, recent gravesite evidence in both states has shown that school administrators often preferred neither to save the Indian nor the man (138, 139). As discussed in Argument Three, the traumatic health impacts of separation in the form of boarding schools, which in some cases continued to operate into the late 20th century, reverberate today. Child theft also occurred through adoption, and Native children continue to be “horribly overrepresented” in the US foster care system despite the Indian Child Welfare Act (140). Now increasingly under assault by the US Supreme Court, this act was established to provide tribal authority over Native child welfare decisions after it became publicly known in the 1970s that up to one-third of all Indigenous children had been separated from their parents. Assimilation, the broader eliminatory technique of which child confiscation is just one part, is the attempted imposition of a colonial nightmare: “have our settler world, but lose your Indigenous soul” (35). A means of co-opting individuals out of their nations and into the settler society, and thus numerically erasing them from the Indigenous collective, assimilation usually occurs in the form of bureaucratic civil procedures after the demise of the frontier^8^ (45). By absorbing Indigenous people into settler markets and modes of land distribution, assimilation is the most permanent means of achieving settler colonialism’s organizing principle: the dissolution of collectively held Native land.

In the US, assimilation was enforced by an “avalanche of … legislation” after the state refused to make further treaties with Native nations beginning in 1871 (35). Assimilation, reinforced by “draconian Supreme Court judgments which notionally dismantled Indigenous sovereignty and provided for the abrogation of existing treaties, relentlessly sought the breakdown of the tribe and absorption into White society of individual Indians and their tribal land, only separately” (35). Achieving elimination through private property, the US divided tribal land into individual allotments, which led to the reduction of Native land by two-thirds in just a half-century. Such a rapid, extreme process of sovereignty disavowal by the settler state left no opportunity for adjustments or an existence consistent with complete health and wellbeing. The goal was to destroy *tribes*, as whole entities, while recruiting *individuals* into settler capitalist society. In the post-World War II civil rights landscape, a similar logic was retained in US policies such as termination and relocation: in the decade following the 1953 legislation, more than 100 Native nations were stripped of tribal status along with 2.5 million acres of land (141). The forcible allotment of Native land was only halted in 1974 with the resistance of the Red Power movement (142). While some forms of coercive assimilationist policies are losing favor, the uneven transition to settler state recognition of Indigenous calls for self-determination has tended to “reproduce the very configurations of colonialist, racist, patriarchal state power that Indigenous peoples’ demands for recognition have historically sought to transcend” (32). In other words, even the most progressive overtures from settler states, in the form of limited acknowledgment and recognition, continue to replicate conditions that are antagonistic to mental and physical health. The existing literature on the health impacts of assimilationist policies and practices, along with other strategies of elimination, is reviewed in Argument Three.

### Argument ii. The settler colonial construction of race

“Beneath the indeterminate signifier of color lies the historical continuity of dispossession, an irregularity that the inclusive regime of race has sought to neutralize.”

-Patrick Wolfe (2016) (45).

“In addition to benefiting from [Indigenous] dispossession, White settlers also benefit from race, the two colonial privileges being fused and mutually compounding in social life. For all their operational cohesion, however, the two are categorically distinct…they can also take separate trajectories.”

-Patrick Wolfe (2016) (47).

Paradies’ worthy question – is invoking colonialism additive to frameworks examining the impacts of racism on health? – challenges us to consider what benefit there is in pursuing one structure’s relationship to health when the other’s, dialectically linked with the first (143), is already so encompassing and relatively well-described. Indeed, despite including papers explicitly referencing the health impacts of settler colonialism and land theft, a recent landmark *Lancet* special issue subsumes these drivers within the triad of racism, xenophobia, and discrimination, none of which fundamentally calls into question settler legitimacy (144, 145). To address Paradies’ question, we scrutinize the connection between settler colonialism and racism, arguing that rather than obviating the need for the former, the health impacts of both racism and settler colonialism are each better understood and analyzed by making explicit the links and distinctions between the two. While settler colonialism also provides crucial insights into the racialization of non-Indigenous populations (141), the unique form of Indigenous racialization within an eliminatory program is only legible, and thus more confrontable, as part of a settler colonial framing. For this reason, Indigenous studies scholarship has historically kept its focus on settler colonialism at a distance from the study of systemic racism, which has sometimes neglected the “genocide of Indigenous peoples as formative in the analysis” (146). In the US in particular, tensions continue to exist in theorizing the racial intersections of settler colonial genocide and slavery (143), while new paradigms are emerging that might unwind them (147).

Description and analysis of the relationship between race and health has a long history in settler colonies (11, 16), even if these polities are generally not described as such in their own histories and mythologies (51). While racialization does not inevitably require a colonial relationship to manifest, this mode of domination is a primary determinant for much of the globe. Race became the “organizing grammar” of colonialism in the industrial age (148), justifying enslavement, appropriation, and exploitation, and it continues to rationalize and reify the manufactured hierarchies of colonial past and settler present. While the process and forms of racialization are heterogeneous and dependent on specific historical context, the overarching goal is the material benefit facilitated by White supremacy. The modern understanding of race emerging in the late 18^th^ century and hegemonic today has two general characteristics: it is hierarchical, with the understanding that distinction from Whiteness implies inferiority, and it essentializes difference by linking “physical characteristics to cognitive, cultural, and moral ones” (45). For all the attempts of liberal multiculturalism to assert that racial difference is value-neutral, this foundational characteristic of race explains why racism as a concept is ultimately redundant: “race already is an ‘ism’” (45).

Analyses from numerous scholars unveil how racialization is itself a product of the settler colony, that races are made in the targeting of people for their land or for their labor, co-constituting Whiteness in the process (34, 35, 44, 116, 119, 131, 141, 149, 150). When the threat of shared social space with the colonized confronts the colonizer, racialization emerges to reinforce hierarchical relations (45). Regarding interpersonal and institutional discrimination in the settler colony, racism is therefore a broad resultant mechanism influencing health outcomes, but the logic behind the discrimination can vary for differently racialized populations. Rather than the diminishing returns of yet another massively encompassing upstream lens, then, settler colonialism enhances the explanatory power of racism as a health determinant by illuminating how and why races are socio-historically fabricated. This in turn clarifies that racism is not a singular force experienced equally across diversely racialized populations. The health manifestations of anti-Black racism will be different from those of anti-Asian racism or antisemitism, and so on. In spite of White supremacy, a number of examples exist in the US of White *inferiority* in health outcomes relative to some other racialized groups (e.g., infant mortality, mental health), further highlighting the health-related importance of racialization’s different manifestations (151).

An example of differential racialization within the US highlights settler colonialism’s explanatory value (34, 44, 45). While racial discrimination is common to both Black and Indigenous populations in the US (44), the modes of domination that generate such discrimination are substantially different, with diverse consequences for racialization and plausible approaches to rectification. The targeting of Africans for labor in the form of chattel slavery led to their becoming the most valuable of any American commodity. This encouraged an inclusive form of racialization, eventually codified in post-emancipation anti-miscegenation laws (i.e., the “one drop rule”) that meant a person was Black as long as African ancestry was present and regardless of phenotypic presentation. The colonizers’ goal was to maximize profit by increasing the number of enslaved people. Antithetically, because Native people were targeted for their land and marked for elimination, their Indigenous status was deemed fragile through tribal status-disqualifying miscegenation, eventually codified in blood quantum laws that were widely adopted as a result of the 1934 Indian Reorganization Act. In other words, unlike Black Americans in the US racial structure, Indian status was considered compromised through intermixing – just as “Black” Aboriginal people in Australia could become “White” in just a few generations (45). What is determinant here is the motive for human targeting, not skin color.

In an exclusive regime of race, Native people are racialized to disappear. For health research, this eliminatory racialization scheme complicates attempts at analyzing outcomes, which require the specific and accurate enumeration of Indigenous people as such (152). By relegating Indians to victims of change rather than subjects capable of modernity, as O’Brien explains, the “degeneracy narrative marred by racial mixing and cultural loss” pushed by White settlers trapped Indians within a schema of erasure whereby any form of change reduced their “Indianness” and any priority claims that might follow (34). These racializing patterns persist beyond North America: as Tatour documents, even well-meaning efforts in support of the Indigenous rights of Palestinian Bedouin have portrayed them as premodern and endangered, operating “as a site of subjectivation that reproduces the racializing logics of settler colonialism and racial imageries of indigenous peoples” (153).

Where the two disparate forms of racialization cohere is in their usefulness to capitalist profit and White supremacy. In brutally removing the Cherokee, Creek, Choctaw, Chickasaw, and Seminole nations in the Deep South, White settlers freed up private land worked by enslaved Black people to produce lucrative cotton (35). Thus the full scope of anti-Black racism in the US cannot be fully appreciated without understanding the state’s position, not only as a vital organ of globally operative racial capitalism (41, 96) but as the world’s first settler colonial state (154). The converging purpose of these divergent racialized histories continues to operate in structural White supremacy today. But because these forms of racialization are so different, even opposite in their original formulations, racism as a single construct cannot fully encompass both when it comes to health and other ramifications. Racism—like race—is contextually heterogeneous.

According to Mamdani’s formulation, because specific colonial agendas meant Africans were enslaved as individuals and Indians were colonized as peoples (44), racism remains primary in evaluating the fortunes of the former and settler colonialism those of the latter. In clarifying between inclusive and exclusive regimes of race, settler colonialism unveils the contours of anti-Indigenous racism, whether in its eliminatory-assimilationist forms in the US or Australia, or in the “racial palestinianization” extant in Israel (116, 119, 155). Appropriately then, the authors of a recent study suggesting that nearly half of the outcome gap in psychological distress between non-Indigenous and Indigenous Australians is due to racial discrimination *also* claim, rather than singling out racism alone, that “inequities are due, at least in large part, to the historical and ongoing effects of settler colonialism and racism” (156). Utilizing only an inclusive understanding of race, as pertains to anti-Black racism in the US, obscures the historical continuities of land-based dispossession and erasure at the heart of Indigenous racialization. Such discourses can therefore reinforce the original colonial injury and ongoing eliminatory logic by offering Native incorporation as the remedy for settler domination (52). The relevance for interventions could hardly be greater, as societal integration can simultaneously be viewed as a *victory* against anti-Black racism and an eliminatory *defeat* for Indigenous people resisting ongoing settler colonization.

This crucial distinction should be reflected in scholarship on health determinants. To group Indigenous people together with other oppressed, minority, or even franchise-colonized populations in invoking racism as the primary culprit is to obscure their different relationships to White supremacy, a distinction with ramifications for understanding health impacts. As discussed in Argument One, certain manifestations and determinants are better understood as Native-eliminatory than as solely the result of racism (28). Wolfe provides such an example from Australia, noting that “while the policy of child abduction was expressed in the language of race…, the Stolen Generations were the centerpiece of a comprehensive campaign that strove for the elimination of an entire group whose definition, as we have seen, is historical rather than biological” (45). This approach signals an epistemic shift from ahistorical bromides lauding a multicultural “nation of immigrants” (96) to a focus on triads of Native-settler-slave/arrivant (52, 131) and Native-settler-immigrant/“undesirable exogenous other” (53, 141). Subsuming Indigenous eradication and its ongoing health impacts into mainstream racial discourse implicitly upholds a liberal multiculturalism in which no one has distinct sovereignty claims, a recapitulation of Indigenous erasure in narrative form. While applying the concept of racism evenly across racialized groups obscures its differing contextual origins and manifestations, the settler colonial lens offers a historicized corrective that can improve our understanding of health and wellbeing. The consequences of failing to do so are significant: in Canada, findings from an in-depth interview study suggest that medical educators’ inability to critically engage questions of race and racialization within a settler colonial context re-entrenches “anti-Indigenous racism and settler dominance” (54).

In medicine, the best treatments are often possible only when we understand *why* a disease is manifesting: what is the underlying cause? This comprehension lends itself to a more targeted, and ultimately successful, treatment approach. Similarly, understanding the drivers and goals of racialization provides us with better tools to challenge its harmful effects. Because race is historically contingent, it is mutable. Gilmore notes that racialization is, by definition, a shifting phenomenon: “racist ideological and material practices are infrastructure that needs to be updated, upgraded, and modernized periodically” (61). The tendency of race to change can be seen in a variety of examples, including the Irish transition to Whiteness (157), the deracination of Arab Jews (45, 158), and Black racialization following the US Civil War. With White power unrestrained after a brief Reconstruction, Black Americans became targeted as surplus in a manner more similar to Native peoples, no longer afforded the protections of bare life that generally if not invariably operated during prior property status. This transition sheds important light on Jim Crow White terror and lynching as primarily *post*-emancipation phenomena (35), as well as the present day mass incarceration, unchilding, and segregated ghettoization that disproportionately impact Black people and epitomize the eliminatory sequestration technologies that were innovated to contain American Indians. Structuring the investigation of racism’s health impacts in ways that incorporate *why* racialization is occurring in particular contexts and with specific manifestations, and distinguishing which elements of racial targeting are better classified as settler colonial elimination, can only enhance our understanding of the determinants of health. For these reasons, a body of literature linking settler colonial manifestations to health outcomes, intersecting with but also distinct from the literature on racism and health, is overdue.

### Argument iii. Future directions and forerunners

“[I]t seems that the settler-colonial analytic must also include reading silence in both the physical sense of indigenous elimination as well as in the suppression and purposeful absence of indigenous voices.”

-Rana Barakat (2018) (159).

The links between settler colonialism and health remain mostly theoretical to date. In Argument One, we discuss some of the specific, present manifestations and technologies of settler colonial elimination that can be analyzed for health impacts. In questioning whether this is a viable approach, we need only look to the analytic framework of structural racism, where conceptual gains linking it to health are accelerating. As has happened with settler colonialism in the last two decades, consolidation around a shared definition of structural racism is moving forward (11, 160). The health impacts of individual components of structural racism, such as redlining and ongoing housing discrimination in the US (161–165), are increasingly elucidated. But new methodologies are also emerging to meet advancements in our understanding of structural racism’s insidious and multifaceted components. Historically, the literature has focused on racism as a driver of social determinants of health, which could then be studied separately but without offering clear or definitive epidemiologic linkages to racism itself. This initial conceptual necessity, similar to what we posit in the introduction regarding settler colonialism as the ultimate organizer of structures and thus social determinants, has limitations when it comes to evaluating causality.

But new approaches to structural racism include both methodologic and analytic innovations, from linking “interdisciplinary variables and data sets and leveraging mixed-method and life-course approaches” for the former, to integrating mixed data and utilizing multidimensional models and latent variable approaches for the latter (166, 167). Empirical studies, making up less than 1% of the literature on racism’s effects on health (167), are now being undertaken in ways that explore “the distal, structural contexts at the root of racialized inequities in health” (167) by utilizing multiple measures (e.g., housing, education, economic, judicial) rather than just a single variable (168). Instead of viewing them only as “ever-more sophisticated critical methodologies,” (79) these approaches spring from a radically transformative position that presupposes histories of colonialism and racism as fundamental drivers of present health inequities. Such emerging methodologies and analytics provide a roadmap to likewise explore the insidious and multifaceted components of settler colonialism, which also traverses systems and sectors across time and in the present.

While few empirical studies have been undertaken, primarily theoretical investigations linking settler colonialism to Indigenous health outcomes have begun, birthing a nascent but powerful literature that provides evidence for the viability of this approach in prospective investigation. And now that settler colonialism has been heavily theorized, opportunities exist to incorporate prior health research that, while not explicitly drawing on settler colonial theory, analyzes some of its specific strategies and manifestations. For a dramatic example, one need only turn to the Indian Health Service sterilization programs of the 1970s. Investigated by Native physicians and the US General Accounting Office, the government eventually admitted that it sterilized thousands of Indigenous women through coerced, unauthorized, and unconsented procedures within just a few years (169). Studies suggest that as many as a quarter to one half of Native women of childbearing age underwent such sterilization procedures, an overtly eliminatory process that saw a dramatic reduction in birth rates across the 1970s, dropping in some communities to levels below that necessary for population maintenance (170). A similar eliminatory logic was reflected in a recent statement by the head of cardiothoracic surgery at a major Israeli teaching hospital, suggesting that Palestinian women who are citizens of Israel should be fined after a certain number of births as a form of Native population control (171).

Evidence already exists to show that displacement, one of the most notable settler colonial strategies of elimination, is bad for health. With 98.9% of their land appropriated as of 2021, Indigenous Americans face an associated increase in exposure to the health effects of climate change, including extreme heat, less rainfall, and reduced food and health security (172). Studies by Saabneh and Daoud and colleagues highlight that, for internally displaced Palestinians in Israel, forced displacement impacts social mobility through reduced educational and vocational opportunities and leads to significantly inferior self-reported health status (173, 174). Yacobi demonstrates that Israeli urban and regional planning impacts access to health services for Palestinian Bedouin through the production of a highly-constrained settler colonial geography, and that by creating a feedback loop of stigma vis-à-vis health and space, territorial control is justified and enhanced (80). Eklund and Martensson utilized geographical information systems to better understand how checkpoints and roadblocks in the occupied West Bank impact healthcare accessibility, highlighting a technology that could be harnessed to understand how displacement and other forms of settler colonial territorial control impact health access (175).

While *immigrant* assimilation into dominant settler society may not have overt health implications (176), Indigenous assimilation’s health impacts have been disastrous if rarely studied in a causal manner. Processes of settler colonial violence and assimilation have decimated Indigenous healing culture and practices while radically shifting dietary possibilities through stolen hunting and farmlands with attendant ruin of traditional healthy means of living (177). Elias and colleagues examined whether direct or indirect exposure to Canada’s Indigenous residential schools, a form of coercive assimilation described in Argument One, is associated with trauma and suicidal behavior. A rare empirical study of settler colonialism’s health impacts, the researchers found that multigenerational exposure to residential schooling was associated with approximately double the risk of abuse, suicidal thoughts, and suicide attempts (178).

The imposition of settler capitalist policies and practices has generated a perverse food economy in which the healthiest foods are the most expensive and unavailable (179), generating remarkably different chronic disease burdens across settler state borders for Native communities like the Pima. Despite dubious reference to susceptible genetics and the lack of terminological specificity in the proposed driver of “Westernization,” researchers identified a much lower prevalence of diabetes in Pima Indians living in Mexico than in the pioneering settler state of the US (180). Few diseases so clearly mark histories of oppression as diabetes, which has been implausibly assigned as a geneticized plague to a variety of racialized groups over the years, from Jewish to Black to Mexican to Indigenous peoples (181). Even after they were corralled into reservations, diabetes was nearly unknown among the Missouri River Indian tribes until they were forced to shift from subsistence methods to dependence on settler dietary commodities in response to a mid-20^th^ century damming project (125). A similar pattern is seen among Palestinian citizens of Israel experiencing land theft and alienation, water confiscation, and forced urbanization (182). While resolutely unassimilated, displaced Palestinian refugees in the captive economy of the West Bank have also experienced new diabetes burdens that, while exceptionally high, are also controllable under conditions of organized refugee-led community health programming and resistance (183, 184). Recent evidence suggests that land theft, deforestation, and the associated urbanization of Indigenous Brazilians are associated with higher rates of obesity and hypertension. Importantly, in terms of disentangling Indigeneity from settler colonial technologies of displacement, these findings did not occur among already-urbanized Brazilians nor among nondisplaced Indigenous Brazilians (185).

Settler colonial strategies of elimination continue to be theorized and formulated, perhaps none as powerfully as Nadera Shalhoub-Kevorkian’s concept of unchilding (75). Unchilding is the process by which colonized children are exposed to forms of violence designed to direct, govern, and racialize them into dangerous others who can then be evicted from childhood and subjected to the full violence of the settler colonial regime. The resonance of this conceptualization reaches beyond Palestine/Israel to states like the US, in which settler police and society frequently dehumanize and unchild Indigenous and Black children (186), easing their physical elimination (187). Beyond violent death, the health consequences of unchilding are numerous and intertwined with other social and eliminatory determinants (71). A number of tools connecting settler colonial social and structural formations to health outcomes have also been proposed, including those designed to capture historical trauma, loss, humiliation, distress, and social suffering (87, 109, 188–191). Systems thinking and complexity theory have been employed to interrogate Indigenous health inequity and narrative accounts amidst prevalent settler colonial logics and “structures of indifference” (70, 84, 192), and linkages between settler colonialism and the transformation of the natural environment, flora, and fauna have been used to explicate racialized infectious disease burdens (193).

In addition to the existing theoretical and empirical evidence for settler colonialism’s negative health impacts, the role of Native resistance in countering these effects is increasingly examined. Palestinian Bedouin have engaged in various creative and legal strategies to remain on their lands and maximize communal health access despite Israeli state plans for their displacement (133). Traditional living and subsistence practices among American Indian and Alaskan Native peoples have positive impacts on mental health and wellbeing, in part through countering social isolation (194, 195). In another study, researchers found that the more cultural factors are present in Indigenous life, including the pursuit of land claims to counter settler colonial dispossession, the lower the risk of suicide among Native youth (196). Across settler state contexts from Oceania to the Americas and in between, food sovereignty among Māori, First Nations, Palestinian, and other Indigenous communities is a growing area of interest in defying the ongoing impacts of settler colonialism (1, 197, 198), and Indigenous scholars continue to push for collective approaches to resilience and resistance rather than the individualistic focus of settler discourse at state and academic levels (199). Finally, challenges to Indigenous data genocide are increasingly mounted, including through the settler colonial reframing and reevaluation of prior studies. In exposing the research that spawned the popular narrative of White disadvantage in “deaths of despair” as reliant on the data elimination of Native people—whose suffering from such deaths and illness is far more extreme—Friedman, Hansen, and Gone also provide guidelines for preventing similar Indigenous data erasures in future research (152).

**BOX 1: Summary of arguments for employing a settler colonial determinants of health framework.**

1) Settler colonial manifestations, techniques, and technologies exist in the present, and their health impacts can therefore be measured. Well-founded causal assumptions are necessary to explain health outcomes, and the logic of elimination provides a guidepost to determine the technologies and techniques that are best understood as settler colonially derived.
2) Racism, on its own, is an inadequate lens for understanding and evaluating Indigenous health inequity in settler colonial contexts. Racialization discourses that do not account for Indigenous genocide, Native sovereignty, and settler colonialism’s ongoing logic of elimination can generate “antiracist” solutions that reinforce colonial injury and its harmful health effects rather than their remediation.
3) Methodologic and analytic innovations in structural racism research can be harnessed to investigate the settler colonial determinants of health. Scholars, predominantly Native, are increasingly utilizing a settler colonial lens as the most appropriate for framing, evaluating, and developing interventions to address Indigenous health.

## Discussion

### Settler decolonization and health

“Settler colonialism has conditioned not only Indigenous peoples and their lands and the settler societies that occupy them, but all political, economic and cultural processes that those societies touch. Settler colonialism directly informs past and present processes of European colonization, global capitalism, liberal modernity and international governance. If settler colonialism is not theorized in accounts of these formations, then its power remains naturalized in the world that we engage and in the theoretical apparatuses with which we attempt to explain it.”

-Scott Lauria Morgensen (2011) (49).

As a result of the remarkable growth of settler colonial studies and its penetration into numerous fields, “[s]ettler colonialism can now be seen where it had not previously been detected” (48). In order to further denaturalize its power, this visibility should be an aspiration for the field of public health. If Morgensen is correct in framing settler colonialism’s reach as transgressing the borders of settler states (49, 200), then its impact on health is even more extensive than we suggest here. To begin building out a literature of settler colonial determinants of health, we have discussed some of the eliminatory manifestations and technologies to analyze, including ongoing frontier violence, displacement, spatial sequestration, various forms of assimilation, sterilization, and unchilding. Others must be named and theorized. Causal diagrams should be carefully built and expanded (201). Given that settler colonialism shapes racialization in the relevant polities, the settler colonial lens offers additional insights into how racism operates and evolves in different contexts and for disparate groups, and with varying health impacts as a result. These distinctions are crucial for the pursuits of health and thriving. Forerunners in exploring settler colonialism’s health impacts have begun making their case through theoretical and empirical work, and scholars in the study of structural racism are building roadmaps that can be borrowed and modified to quantitatively analyze the multifaceted manifestations of the settler colonial present.

The absence of discourse on settler colonialism in the fields of public health and medicine, at least until very recently, highlights a conceptual gap impeding progress toward a health equity that refuses to substitute the settler biopolitical regime’s focus on “a proportionate distribution of health and disease” for Native decolonial approaches with transformative potential (202, 203). Through further theorizing and empirical study, settler colonialism must be better described and challenged in the future if this goal is to remain in sight. Even if one remains unconvinced that this framework adds to rather than simply complexifying the analysis of racism’s health impacts, prescriptions to eliminate or even ameliorate racism may be doomed without its dismantling. Because differential racialization reflects differential colonization or marginalization, antiracism agendas without decolonization are likely to remain, at best, incomplete. If settler colonialism, the land-based project of accumulation through dispossession, drives much racialization, then dismantling structural racism requires nothing less than the transformation of its dialectical counterpart (149). And if decolonization is necessary to end franchise colonialism and to achieve the full spectrum of sovereignty and health in such contexts, this is as true for settler colonialism and the associated need to address, and indeed redress, racial health inequities for Indigenous and non-Indigenous racialized communities. Just as the WHO has declared apartheid – a specific structure of racialized settler colonial rule (135) – incompatible with the fulfillment of the right to health (136), settler colonialism itself remains an impassable and ongoing impediment to this right.

While massive settler exoduses have occurred in response to Native resistance, as in the case of the French settlers in Algeria, decolonizing settler colonialism represents a more enduring and entangled challenge than that of franchise colonialism. A return to pre-settler life is no longer possible, and in most cases the settlers, often many generations into the colonial project, no longer have another state or homeland to which they can return. Still, decolonization demands the repatriation of Indigenous lands and life (131), requiring the restitution and reparations that are increasingly shown to alleviate health inequities (204–207). But it also entails much more than acknowledgment, recognition, and restitution because settler colonialism is not a *past* event to reconcile. Undoing a living structure is more arduous and ongoing work (53). Decolonizing the political apparatus via a constructive reparatory program is particularly important in settler colonial states where the revolutionary path is less clearly marked (208). This entails the development of a political community that rejects colonial categories, emphasizes and rectifies power asymmetries, and removes the nation as the locus of political identification, thus eradicating concepts of permanent majorities and minorities within the political sphere and opening the possibility of emancipation for Indigenous and settler alike (44, 56, 209). How precisely this process might intersect with and elevate Indigenous sovereignty is not yet fully clear or excavated, but Indigenous activists and scholars are charting politics of refusal (62), resurgence (210, 211), survivance Land Back (212, 213), environmental repossession (214), grounded normativity (32, 215), environmental defense (216), therapeutic reclamation (217), bad biocitizenship (202), extra-colonial visioning (218, 219), *sumud* (220), culture-centred approaches and solidarities (221–222), embodied resistance (223), unrelenting anticolonial struggle (63), and the dismantling of supremacist knowledge production paradigms (57), each of which brings us closer.

It is this last objective, to challenge the epistemicide inherent to Western modernity (98, 224, 225), that must infuse study of the settler colonial determinants of health. Required is a solidarity-based approach that integrates Indigenous studies and diligently avoids becoming a “largely White attempt to think through contemporary colonial relationships” (226). Beyond individual endeavors, this demands a growing movement of health scholars seeking to delineate the ongoing depredations of settler colonial states alongside the attainments of Indigenous resistance, with the goal of transforming our understanding of the linkages that make some more likely to get sick and die than others. Such an approach, demanding the utmost scholarly rigor, is no more or less political than those currently hegemonic. It is from this vantage point, and as the leadership collective of the Palestine Program for Health and Human Rights, that we offer the settler colonial determinants of health as an analytic framework ripe for questioning, studying, and challenging health inequities.

## Data availability statement

The original contributions presented in the study are included in the article/supplementary material, further inquiries can be directed to the corresponding author.

## Author contributions

BW conceived the theoretical approach and drafted the manuscript. DM, OT, YA, and WH contributed to the conception of ideas and structure. DM contributed background literature review. All authors contributed to the manuscript revision, read, and approved the submitted version.

## Contributions to the field

This manuscript introduces an analytic framework for research, pedagogy, and interventions regarding Indigenous health in settler colonial contexts, drawing extensively from the social science and public health literatures. While it is well described that Indigenous people suffer significant health inequities in comparison with settler counterparts, the mechanisms for these disparities remain obscure and partial. We argue that settler colonial theory provides a means to rectify these deficits in understanding, similar to the role critical race theory has played in the development of methodological and analytic tools to study the health effects of structural racism. Our three arguments are that 1) settler colonial manifestations are ongoing and thus can be prospectively investigated, 2) that settler colonialism enriches yet remains distinct from understandings of racism as a health determinant, and 3) that new theoretical and empirical studies and methodologies are now available that suggest the viability of this approach in public health research and action. While much additional work remains, this Hypothesis/Theory paper presents an initial offering of an analytic intended for broad use in evaluating research questions and interventions to address Indigenous health outcomes and inequities.

## Funding

The Palestine Program for Health and Human Rights at the FXB Center for Health and Human Rights contributed open access publication fees.

## Conflict of interest

The authors declare that the research was conducted in the absence of any commercial or financial relationships that could be construed as a potential conflict of interest.

## Publisher’s note

All claims expressed in this article are solely those of the authors and do not necessarily represent those of their affiliated organizations, or those of the publisher, the editors and the reviewers. Any product that may be evaluated in this article, or claim that may be made by its manufacturer, is not guaranteed or endorsed by the publisher.

## Footnotes

^1^Furthermore, this article would not exist, at least in its current form, without the probing and engaged student and guest speaker discussions from our multidisciplinary graduate seminar, *The Settler Colonial Determinants of Health*. The course, and by extension this paper, has already expanded and improved through student feedback and communal class attempts at refining what a settler colonial health framework can and should entail, and how it can be operationalized in empirical research. In this important sense, the article reflects ideas and discourse from a collective pedagogical endeavor to engage a new framework that might provide improved understanding of what drives, and how to transform, specific health inequities.

^2^While we take the position that all historic Palestine is under settler colonial control, we use here a dual name for the land, both the historic name and that of the settler colonial state, due to the lack of clarity regarding how much of the territory is currently considered Israel.

^3^Following Glen Coulthard (32), we use the terms Indigenous, Native, and Aboriginal interchangeably to describe descendants of those who traditionally occupied their respective territories before the arrival of European settlers and their eventual state powers; we additionally use the term Indian, following Jean M. O’Brien (34), for the peoples in what became the United States.

^4^Sometimes termed exploitation colonialism or just colonialism.

^5^Where settler colonialism is structurally ongoing, as in the majority of contexts, settler status continues to pass across generations.

^6^In 1923, Cayuga leader Deskaheh succinctly anticipated both the logic of elimination and the observation that “settler colonizers come to stay” many decades before Wolfe’s famous formulations: “The governments at Washington and Ottawa have a silent partnership of policy. It is aimed to break up every tribe of Redmen so as to dominate every acre of their territory. Your high officials are the nomads today – not the Red People. Your officials will not stay home” (78).

^7^This point pertains regardless of whether the settler context and strategy has shifted from significant levels of unconcealed state or mob coercion and violent displacement to predominantly bureaucratic procedures and the “organized violence” of police control and incarceration (32, 61).

^8^Notably, a still-open frontier, racial exclusion, and persistent resistance make Palestinians largely unassimilable within settler society today (71).
